# Temporal Pitch Sensitivity in an Animal Model: Psychophysics and Scalp Recordings: Temporal Pitch Sensitivity in Cat

**DOI:** 10.1007/s10162-022-00849-z

**Published:** 2022-06-06

**Authors:** Matthew L. Richardson, François Guérit, Robin Gransier, Jan Wouters, Robert P. Carlyon, John C. Middlebrooks

**Affiliations:** 1 Department of Otolaryngology, Center for Hearing Research, University of California at Irvine, Irvine, CA USA; 2 Departments of Neurobiology & Behavior, Biomedical Engineering, Cognitive Sciences, University of California at Irvine, Irvine, CA USA; 3 Cambridge Hearing Group, MRC Cognition and Brain Sciences Unit, University of Cambridge, Cambridge, UK; 4 Department of Neurosciences, ExpORL, KU Leuven Leuven, Belgium

**Keywords:** temporal pitch perception, acoustic change complex, frequency following response, cochlear implant, cat, harmonic complex

## Abstract

Cochlear implant (CI) users show limited sensitivity to the temporal pitch conveyed by electric stimulation, contributing to impaired perception of music and of speech in noise. Neurophysiological studies in cats suggest that this limitation is due, in part, to poor transmission of the temporal fine structure (TFS) by the brainstem pathways that are activated by electrical cochlear stimulation. It remains unknown, however, how that neural limit might influence perception in the same animal model. For that reason, we developed non-invasive psychophysical and electrophysiological measures of temporal (i.e., non-spectral) pitch processing in the cat. Normal-hearing (NH) cats were presented with acoustic pulse trains consisting of band-limited harmonic complexes that simulated CI stimulation of the basal cochlea while removing cochlear place-of-excitation cues. In the psychophysical procedure, trained cats detected changes from a base pulse rate to a higher pulse rate. In the scalp-recording procedure, the cortical-evoked acoustic change complex (ACC) and brainstem-generated frequency following response (FFR) were recorded simultaneously in sedated cats for pulse trains that alternated between the base and higher rates. The range of perceptual sensitivity to temporal pitch broadly resembled that of humans but was shifted to somewhat higher rates. The ACC largely paralleled these perceptual patterns, validating its use as an objective measure of temporal pitch sensitivity. The phase-locked FFR, in contrast, showed strong brainstem encoding for all tested pulse rates. These measures demonstrate the cat’s perceptual sensitivity to pitch in the absence of cochlear-place cues and may be valuable for evaluating neural mechanisms of temporal pitch perception in the feline animal model of stimulation by a CI or novel auditory prostheses.

## Introduction

Many aspects of human speech and animal vocalizations consist of pitch-evoking harmonic tone complexes that repeat at a rate corresponding to the fundamental frequency (F0). Normal-hearing (NH) human listeners can extract pitch from this F0 periodicity even when the sound contains only high-numbered harmonics that are not resolved in frequency by the peripheral auditory system (Hoekstra 1979; Houtsma and Smurzynski 1990; Shackleton and Carlyon 1994). Cochlear implant (CI) users can detect F0 periodicity either from amplitude modulations applied to fixed-rate electric pulse trains (Wouters et al. 2015) or from the temporal fine structure (TFS) of single-pulse-per-period pulse trains (Shannon 1983; Townshend et al. 1987). Nevertheless, CI users generally show a much narrower range of discernable pulse rates than do NH listeners. For example, typical CI users have an upper limit of about 300 pulses per second (pps), above which they are insensitive to changes in pulse rate (Carlyon et al. 2008; McKay et al. 2000; Shannon 1983; Tong and Clark 1985; Townshend et al. 1987; Zeng 2002). In contrast, NH listeners are sensitive to temporal pitch produced by unresolved harmonic complexes at rates up to 700–800 pps (Carlyon and Deeks 2002; Macherey and Carlyon 2014) and TFS of pure tones at least up to ~ 2000 Hz (Moore 1973; Verschooten et al. 2019).

Single-unit recordings in cats show that temporal sensitivity to CI stimulation is limited, in part, by poor transmission of TFS at the level of the brainstem (Snyder et al. 1995, 2000; Vollmer et al. 1999, 2005; Shepherd et al. 1999; Hancock et al. 2013; Middlebrooks and Snyder 2010). Specifically, Middlebrooks and Snyder (2010) showed in acutely deafened cats that the upper limit of neural phase locking in the inferior colliculus to electric stimulation was low (< 300 pps) for conventional intracochlear electrodes positioned in the basal cochlea (i.e., stimulating high-frequency pathways) but increased to about an octave higher for penetrating auditory-nerve electrodes that selectively stimulated fibers from the low-frequency cochlear apex. In both cases, this upper limit declined dramatically with longer durations of deafness (Middlebrooks 2018). The perceptual relevance of these brainstem limitations remains unknown, however, inasmuch as purely temporal pitch sensitivity in behaving cats has not been evaluated even for normal hearing, let alone electric hearing. Moreover, we do not know the level of the auditory pathway that is responsible for the limitations in temporal pitch perception with CI stimulation, either in cats or in humans, nor do we fully understand the influence of the history of deafness and electrical stimulation on the degradation of temporal acuity. For those reasons, we first aimed to characterize temporal pitch sensitivity in NH cats presented with band-limited acoustic pulse trains that restrict stimulation to the basal cochlear ranges that are stimulated by CIs. Second, we aimed to develop non-invasive objective measures that can relate the cat’s perceptual sensitivity to temporal processing at various stages of the auditory pathways.

In perceptual measures, we trained NH cats to detect changes in the rates of acoustic pulse trains in a psychophysical task that assessed their sensitivity to changes in temporal pitch. In objective measures, we evaluated scalp recordings of the acoustic change complex (ACC) and frequency following response (FFR) for the same stimuli in sedated cats. The ACC is a cortical potential evoked by various changes in an auditory stimulus (e.g., frequency, timing, level; Martin and Boothroyd 2000). In humans, threshold stimulus changes for the ACC have been shown to correspond well with perceptual difference limens, both for NH (Martin et al. 2010; Han and Dimitrijevic 2015; He et al. 2012; Guérit et al. submitted) and CI listeners (Han and Dimitrijevic 2020; Mathew et al. 2017). In cats, the ACC has been shown to be elicited by changes in pure-tone frequency and level (Presacco and Middlebrooks 2018). The ACC thresholds in that study corresponded well with published cat behavioral frequency and level discrimination. The FFR is a brainstem-generated potential that synchronizes to stimulus periodicity, the strength of which is believed to reflect the fidelity of TFS encoding relevant to pitch processing (Bidelman et al. 2011; Bidelman and Krishnan 2009; Krishnan and Gandour 2009; Krishnan and Plack 2011; Guérit et al. submitted).

We addressed several fundamental questions. Do cats show psychophysical evidence of temporal pitch sensitivity in the absence of place-of-excitation (i.e., spectral) cues produced by resolved frequency components? If so, how does cat temporal pitch sensitivity compare with that of NH human listeners? Is the ACC sensitive to the purely temporal changes in pulse rates? How does its sensitivity compare with the perceptual measures? How does the FFR, a brainstem measure, compare with the ACC (a cortical measure) and with perception? The answers obtained to these questions demonstrate the value of this feline animal model for studying the fundamental basis of temporal pitch perception as well as for practical issues relevant to conventional CIs and novel forms of auditory prosthesis.

## Materials and Methods

### Overview and Stimulus Design

**Fig. 1:**
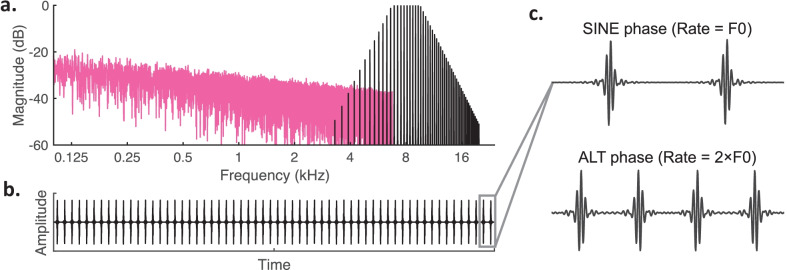
Example of the acoustic pulse train: spectral and time representations. **a** Amplitude spectrum of the bandpass harmonic complex (black lines) centered on 8 kHz, depicted for an arbitrary F0 value. The bandpass cut-off frequencies were always 8 kHz ± ¼-octave (6727.2 to 9513.7 Hz) with 48 dB/octave linear slopes beyond those cut-offs. Pink noise (pink) was used to mask distortion tones arising among harmonics. **b** Temporal pulsatile waveform for the bandpass harmonic complex in (**a**). **c** A segment of acoustic pulses shown for two stimulus designs of the bandpass harmonic complex in (**a**): top: SINE phase, all harmonic components were generated in sine phase and the pulse rate was equal to the F0; bottom: ALT phase, harmonic components alternated in sine phase (odd harmonics), and cosine phase (even harmonics) and the pulse rate was equal to 2 × F0

The psychophysical task and scalp recordings were performed in the same laboratory using a similar stimulus design. Acoustic pulse trains were generated in the time domain by summing a bandpass series of harmonic components limited to harmonic numbers 15 or higher (see Fig. 1a). The spacing of those high harmonics is narrower than the resolvability of the ear, thereby removing place-of-excitation (i.e., spectral) cues in deriving pitch (Shackleton and Carlyon 1994). These stimuli produce steeply rising temporal envelopes that in psychophysical tasks have been shown to provide purely temporal cues resembling those of single-electrode CI pulse trains (see Fig. 1b) (Carlyon and Deeks 2002; Hoekstra 1979; Macherey and Carlyon 2014; Shackleton and Carlyon 1994; van Wieringen et al. 2003). The center frequency of the bandpass harmonic complex was 8000 Hz, which targeted approximately the basal cochlear turn that can be stimulated by a cochlear implant positioned in a cat. The cut-off frequencies were ¼-octave below and above the center frequency (6727.2 to 9513.7 Hz) with 48 dB/octave linear slopes beyond these cut-offs. For a point of reference, we estimated the equivalent rectangular bandwidth (ERB) of the feline auditory filter centered at the lower cut-off frequency of 6727.2 Hz to be 975 Hz, based on notched-noise psychophysical experiments conducted in cats in our laboratory (Guérit et al. 2022a). Continuous pink noise was added to mask distortion tones arising among harmonics. The pink noise level was adjusted to have an 8 kHz spectrum level that was 47 dB below the pulse train root-mean-square (RMS) level (cf. Deeks et al. 2013) and then was low-pass filtered at 6727.2 Hz to minimize overlap with the pulse train passband.

Temporal pitch changes were introduced to the pulse trains by switching between a base pulse rate and a rate that was either ~ 36 or ~ 66 % higher. To minimize loudness differences between the base and higher rates, the higher-rate pulse trains were reduced in level to equalize their RMS levels with that of the base rates. The transition between pulse rates always occurred at the zero-amplitude midpoint between consecutive pulses to eliminate spectral splatter.

Table 1 shows the standard base and higher rates used in both the electrophysiological and psychophysical experiments; supplemental conditions are described in the Psychophysical and ACC results section. All pulse rates were chosen to differ by at least 5 Hz from harmonics frequencies 1 to 12 of 50 Hz (Europe) and 60 Hz (USA) domestic power supplies. The selected range of standard pulse rates was based on NH human performance with these stimuli, which is most sensitive at rates of ~ 100 pps and declines for rates above about 500 to 600 pps (Carlyon and Deeks 2002; Macherey and Carlyon 2014; Stahl et al. 2016). This range also provides a relevant acoustic baseline for electric stimulation, inasmuch as most human CI listeners have poor rate sensitivity above 300 pps (e.g., Carlyon et al. 2008) and cats show limited phase-locking above 200 pps at the level of the inferior colliculus for conventional CI electrodes positioned in the basal cochlea (Middlebrooks and Snyder 2010). Pulse trains with base rates as high as 280 pps (+ 36 % = 380 pps, + 66 % = 464 pps) were generated with all harmonic frequencies in sine phase, which produces a pulse rate equal to the F0. Base rates higher than 280 pps were generated by alternating harmonic frequencies in sine phase (odd harmonics) and cosine phase (even harmonics). This alternating-phase design produces a pulse rate equal to 2 × F0, which helped to prevent spectral resolvability at higher rates by maintaining closely spaced harmonics within the passband (Shackleton and Carlyon 1994). The two designs are denoted respectively as SINE and ALT phase (Fig. 1c).

**Table 1:** Standard pulse rate conditions. Columns specify the exact harmonic complex pulse rates (pps; black text) used for each base-rate condition (top row) and corresponding rate increases of 36 % (middle row) or 66 % (bottom row). The fundamental frequency (F0; italicized text) for each harmonic complex is specified below each pulse rate value. For SINE-phase conditions (left three columns), the pulse rate was equal to the F0. For ALT-phase conditions (right three columns), the pulse rate was equal to 2 × F0

	**Standard pulse rate conditions**					
	SINE phase			ALT phase		
Base rate (pps) *F0 (Hz)*	94 *94*	188 *188*	280 *280*	376 *188*	472 *236*	560 *280*
+ 36 % *F0*	128 *128*	256 *256*	380 *380*	512 *256*	640 *320*	760 *380*
+ 66 % *F0*	156 *156*	312 *312*	464 *464*	624 *312*	784 *392*	928 *464*

### Animals

All procedures were in accordance with the NIH Animal Welfare Guidelines and with protocols approved by the Institutional Animal Care and Use Committee at the University of California at Irvine. Domestic shorthaired cats (*Felis catus*) were obtained from a breeding colony at the University of California at Davis. No hearing deficits were evident.

A total of eight cats were used for the study. Four trained cats (2 female, 2 male) participated in both the psychophysical and electrophysiological experiments. Ages ranged from 3 to 6 months at the beginning of training. After training periods that varied in duration, the reported psychophysical data were collected over a 12-month period during which ages ranged from 7 to 33 months of age. Cats performed in the psychophysical experiment 4 to 5 days a week. On those days, cats received moist food as positive reinforcement during the behavioral task. Cats generally performed the task until sated but were given free access to dry food for ~ 1 h after the session. On days when cats did not perform in the psychophysical experiment, including weekends, they were given free access to dry food for 3 h per day. Water was freely available in the housing area.

Four untrained cats (3 female, 1 male) participated exclusively in the electrophysiological experiment. Those cats ranged from 37 to 59 months of age at the time the reported data were collected. They were given free access to dry food and water in the housing area 7 days a week. All male cats in the study were neutered to reduce aggressive behavior and thereby facilitate group housing.

### Psychophysics: Apparatus and Procedure

The psychophysical experiment was performed inside a double-wall sound-attenuating chamber (Industrial Acoustics, inside dimensions 2.6 × 2.6 × 2.5 m) lined with SONEXone acoustic foam panels. Stimulus generation, experimental control, and data acquisition used System III TDT hardware controlled by custom MATLAB software on a Windows-based desktop computer. Sounds were generated at a sample rate of 97,656 s^−1^ with 24-bit precision. Pulse-train and pink-noise stimuli were presented through a 3″ co-axial loudspeaker (Fostex FF85WK) in a bass reflex enclosure. Prior to each experimental session, the speaker was calibrated in the absence of the cat using a ½-inch precision microphone (ACO Pacific) positioned in the sound field in the normal location of the cat’s head. Calibration probe sounds were maximum-length sequence Golay codes (Zhou et al. 1992). Inverse spectra were derived for the purpose of equalizing sound spectra to within a standard deviation of < 1 dB from 50 to 25,000 Hz at known sound pressure levels re 20 µPa (i.e., re 0 dB SPL). During the experiment, the pulse train stimulus was presented at a level of 60 dB SPL and gated simultaneously with the pink noise.

The cat sat or stood on an elevated platform in the center of the chamber and directly faced the speaker located 1.2 m in front of the cat’s head. A harness restrained the cat to the platform but allowed for free movement of the head and limbs. The cat generally maintained head and pinnae orientation towards the speaker. A response pedal and computer-controlled feeder were mounted to the pedestal in front of the cat. The feeder delivered small portions of liquified commercial cat food as a behavioral reward. The cat performed the task in the dark. The experimenter controlled the task from outside the booth using a graphical user interface and monitored the cat using an infrared video display.

#### Behavioral Task and Training

The psychophysical task was adapted from the hold-release paradigm described by May and colleagues (1995) and implemented successfully in our previous studies (e.g., Javier et al. 2016). To initiate a trial, the experimenter illuminated a green light located near the sound source. The green light signaled the cat to press and hold the pedal, which initiated a continuous pulse train with a given base rate. After a variable hold duration, the pulse train switched to a higher pulse rate and the cat could receive a food reward by releasing the pedal within a criterion window of 600 ms after the rate change. On each trial, the hold duration was randomly sampled from 2.6, 3.2, 3.8, or 4.4 s (i.e., intervals of 600 ms) denoted, respectively, as Hold 1, Hold 2, Hold 3, and Hold 4. The criterion window was shortened from our previous study (1000 ms; Javier et al. 2016) as pilot experiments showed an improved training effect by requiring responses closer to the rate change. The stimulus terminated upon a release of the pedal at any time, or 1200 ms after the pulse-rate change if the pedal was not released.

**Fig. 2:**
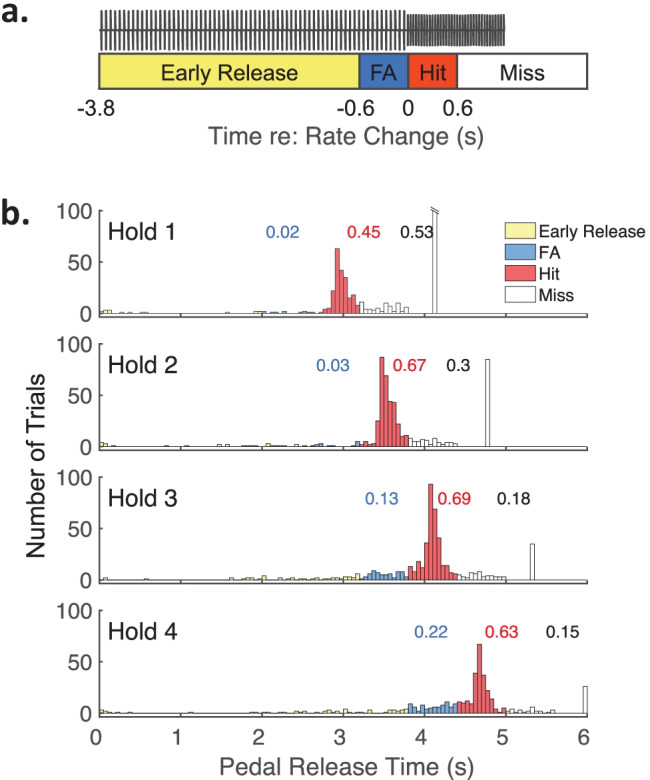
Schematic of the psychophysical paradigm and response latencies for Cat Ha. **a** Example trial of the hold-release task. The cat initiated the acoustic pulse train (black waveform) by pressing and holding a pedal. After 3.8 s (i.e., the hold time), the pulse train increased from a base rate to a higher rate (depicted at 0 s); the higher-rate pulse train was always reduced in level to equalize its RMS level to that of the base rate. The stimulus terminated upon a release of the pedal at any time, or 1200 ms after the rate change if the pedal was not released. Colored boxes show the possible scores on each trial: a release 600 ms before the rate change was an “Early Release” (yellow), a release 600 to 0 ms before the rate change was a “False Alarm” (FA, blue), a release 0 to 600 ms after the rate change was a “Hit” (red), a release later than 600 ms after the rate change was a “Miss”. **b** Response latency histograms for cat Ha combined across all standard base-rate and change conditions. Each of four possible hold times are represented in the 4 panels, where the stated time is the duration of the base-rate stimulus: Hold 1 = 2.6 s; Hold 2 = 3.2 s; Hold 3 = 3.8 s; Hold 4 = 4.4 s; see text for an exception for Cat Ri. Bar colors represent the trial scores as labeled in (**a**). Latencies for misses that occurred after the stimulus offset were not recorded and therefore combined into one (white) bar. Early releases were excluded from measures of performance. For each hold time, blue, red, and black numbers indicate the proportion of trials that were scored as FA, Hit, or Miss, respectively

A pedal release within the criterion window was scored as a “Hit” and elicited a food reward. A release later than 600 ms after the rate change was scored as a “Miss.” A release 600 to 0 ms before the rate change was scored as a “False Alarm” (FA). A pedal release more than 600 ms before the rate change was recorded as an “Early Release” and not scored. Misses, FAs, and early releases resulted in a 2-s time-out period signaled by a flashing blue light. Figure 2a illustrates these time ranges for Hold 3 trials. One cat (Ri) showed a strong bias for longer hold durations but otherwise performed the task well. To accommodate this case, hold times for cat Ri were shifted by 400 ms; Holds 1, 2, 3, and 4 = 3, 3.6, 4.2, and 4.8 s.

Training in the task and data collection varied in duration among cats, ranging from 4 months to a year. Data collection was completed when the cat completed at least 30 trials per hold time (Hold 1, 2, 3, 4 = 120 in total) for each of the 6 standard base-rate and 2 rate-change conditions.

#### Behavior Analysis

Figure 2b shows the pedal-release latencies by one cat for all trials combined across base-rate and rate-change conditions. Early release (bar color: yellow), FA (blue), hit (red), and miss (white) are indicated for each hold time; miss latencies that occurred after the stimulus offset were not recorded and therefore combined into one (white) bar. Note that the false alarm response-time window for Hold N + 1 trials coincided exactly with the hit window of Hold N trials. For that reason, we used the false alarm rate during Hold N + 1 trials as catch (no rate-change) trials for Hold N trials. The absence of a FA during a Hold N + 1 trial (i.e., a Hit or a Miss) meant that the cat correctly rejected the absence of a rate change corresponding to the hit window of a Hold N trial.

Performance was measured by the sensitivity index, *d’*, for each base-rate and rate-change condition.

$${d}^{{\prime}}= z\left({P}_{Hit}\right)- z\left({P}_{FA}\right)$$

The proportion of hits (*P*_Hit_) was computed over Holds 1–3 as the number of trials scored hit divided by the number of all trials scored as hit and miss trials. The proportion of FAs (*P*_FA_) was computed over Holds 2–4 as the proportion of trials scored FA divided by all trials scored FA, hit, or miss. *P*_Hit_ was counted separately for each base-rate and rate-change condition. *P*_FA_ was counted for each base-rate condition but combined across rate changes because the size of the change was irrelevant to a false alarm or correct rejection response made in the absence of the rate change. The *P*_FA_ tended to increase with increasing hold time, which was attributed to the cat’s impatience for a food reward. To mitigate any bias introduced by this trend, the *P*_FA_ was first obtained separately for each hold time and weighted according to the distribution of hit and miss trials at the same hold times, then the weighted average *P*_FA_ was computed; hereafter, the weighted *P*_FA_ is simple denoted by *P*_FA_. *d’* was then computed as the difference of the standard deviants (i.e., z-scores) of all *P*_Hit_ and *P*_FA_.

The threshold for rate-change detection was defined as *d’* ≥ 1. The chance level for *d’* was estimated for each condition by performing a permutation test in which *d*’ was computed after randomizing the experimental hold times with respect to the cat’s actual pedal releases. This procedure was repeated for 1000 permutations, which yielded a distribution of *d*’ values that was typically centered around zero (i.e., no discrimination). The 95th percentile of that distribution was used as a measure of chance performance. Chance values of *d*’ measured in that way were consistently lower than 1. The variability of individual cat performance was estimated for each condition by resampling trials with replacement 1000 times and computing the standard deviation over samples (i.e., the bootstrapped standard error; Efron and Tibshirani 1991).

Criteria for including sessions in the final analysis were established to remove early training sessions or sessions with uncharacteristically poor performance. First, to obtain a sufficient measure of intra-session performance, only sessions with at least 10 scored trials were retained. Second, after combining all trials across base-rate and rate-change conditions, only sessions in which *P*_Hit_ > 0.5 and *P*_FA_ < 0.5 were retained. This criterion required *d’* greater than 0 while screening out highly biased performance; for instance, *P*_FA_ ≈ 0 due to persistent pedal holding would have produced artificially high *d’* values, even for *P*_Hit_ ≲ 0.5.

### ACC and FFR: Apparatus and Procedures

The electrophysiological recordings were conducted in a single-wall sound-attenuating chamber. Stimulus generation and waveform recording used System III hardware from Tucker-Davis Technologies (TDT; Alachua, FL) controlled by custom MATLAB scripts (The Mathworks, Natick, MA) on a Windows-based desktop computer. Acoustic stimuli were generated at a sample rate of 97,656 s^−1^ with 24-bit precision. The pulse train stimulus was presented through a Radio Shack horn tweeter, and the pink noise was presented through a 3-inch co-axial speaker (Pioneer TS-A878), both located 23 cm to the left of the cat’s left ear. The speakers were calibrated prior to each experimental session as described for the psychophysical experiment but with the microphone positioned in the sound field in the location of the cat’s left pinna. During the experiment, the pulse train was presented at 65 dB SPL and was gated simultaneously with the pink noise. It was found in pilot studies that increasing the stimulus level relative to psychophysical procedure (i.e., 60 dB SPL) improved the ACC signal-to-noise ratio. For that reason and given that psychophysical data collection had already begun, a 5-dB level increase was chosen for ACC/FFR stimulation, which still improved the SNR, but did not differ largely from the psychophysical procedure. Note that the behaving cats might have experienced some benefit from binaural summation and some acoustic gain due to the position of the pinnae in the sound field, thereby narrowing the effective level difference from the electrophysiological procedure (Guérit et al. 2022a).

The efficiency of the ACC procedure was enhanced by presenting pulse-train stimuli continuously using an alternating pattern paradigm (e.g., Martin et al. 2010). In that paradigm, the pulse rate always began at the base rate for 1 s, then switched to the higher rate for 1 s, then returned to the base rate, and so on for a total of 12 s per block. Each block therefore contained 6 sweeps from the base- to the higher-rate pulse train. A full run consisted of 25 blocks that were each separated by 2-s silent intervals, which produced a total of 150 sweeps per condition (6 sweeps × 25 blocks). Block onsets were assigned random temporal perturbations of σ = 1 s to minimize possible effects of ongoing oscillations on the averaged waveforms. The total testing duration for each condition was ~ 5.8 min.

Experimental conditions included twelve combinations of 6 base rates with ~ 36 % or ~ 66 % changes, presented in random order across cats (see Table 1). The polarity of the stimulus waveform was inverted for each successive pair of base- and higher-pulse rate to minimize possible electrical artifacts from the stimulus transducer in the averaged FFR waveforms. The total duration of an experimental session was 1.5 to 2 h.

#### Scalp Recording

Cats were sedated using a light level of anesthesia induced with an intramuscular injection of ketamine (20 mg/kg) and acepromazine (1 mg/kg). At those doses, eye-blink or limb-withdrawal reflexes sometimes could be elicited, but there were no spontaneous movements. When necessary, a supplemental dose of ketamine alone was given at least 30 min after the initial injection to maintain an immobile state. Scalp potentials were obtained with subdermal hypodermic needle electrodes. Pilot data with the present stimulus paradigm were consistent with the pure-tone frequency and intensity results of Presacco and Middlebrooks (2018) in showing that the cat’s ACCs were generally larger when recorded from a contralateral than an ipsilateral scalp region relative to the sound source. For that reason, two active electrodes were placed on the cat’s right hemisphere, contralateral to the sound source. Both active electrodes were approximately 1–2 cm from the midline; one was aligned with the center of the pinna and the other was ~ 1 cm anterior to the margin of the pinna (~ 3 cm between electrodes). The reference electrode was placed on the left mastoid and the ground electrode was placed on the back of the cat. Recorded waveforms were amplified with a TDT low-impedance head stage*,* digitized at a sample rate of 24,414 s^−1^ and high-pass filtered at 1 Hz to eliminate DC voltage. The signal was then down-sampled online to 12,207 s^−1^ and stored on a computer.

#### ACC Analysis

Scalp-recorded waveforms were bandpass filtered between 2 and 20 Hz; we used a Butterworth design with the zero-phase (non-causal) MATLAB *filtfilt* function, which yielded a 4th order filter. The two active electrode channels were averaged together for analysis as there were negligible differences in their results. The waveforms were then segmented around 400 ms before to 2000 ms after the onset of each base rate; there were no direct-current corrections of the waveforms. Each waveform epoch therefore contained a full sweep of the base rate (0 to 1000 ms) to the higher rate (1000 to 2000 ms). Waveforms for the first sweep in each block were removed to exclude stimulus onset responses from the ACC analysis. This procedure resulted in 125 waveforms per condition (150 total sweeps – [1 sweep × 25 blocks]). Evoked responses within an analysis window of 15 to 250 ms following the decreasing-rate change at 0 ms and increasing-rate change at 1000 ms were respectively referred to as decreasing-rate ACC and increasing-rate ACC. To screen for excessive noise, individual waveforms for each condition were excluded if amplitudes within the analysis windows exceeded a factor of the RMS obtained within these intervals across all waveforms. To account for varying levels of background noise in the recordings, the RMS factor was adjusted for each cat and experimental session so that the average number of rejections across all conditions was between 4 and 6 %; this resulted in an average of 4.8 % total rejected waveforms.

The remaining ~ 118 waveforms for each experimental condition were averaged. When present, the ACC response consisted of a positive peak (P1), followed by a negative peak (N1). A second positive peak (P2) was sometimes present but was not consistent in morphology among cats and conditions. Based on inspection of the grand mean waveforms across cats, the P1 and N2 were respectively selected as the maxima/minima voltages from 15-to-65 ms and 40-to-130 ms with respect to each decreasing- or increasing-rate change. For each cat and experimental condition, the ACC magnitude was quantified as the peak-to-peak P1 and N1 amplitude difference (i.e., P1-N1). In each of these cases, neural noise floor levels were estimated separately for the selected P1 and N1 by computing the amplitude variance at each peak’s mean latency across trials and dividing by the square root of the number of trials. The ACC noise floor level was the average of those P1 and N1 noise values. Latency values were obtained at both the P1 and N1 peaks and were analyzed separately.

A receiver-operating characteristic (ROC) analysis was used to determine the significance of the ACC magnitudes for individual cats (Green and Swets 1966; Macmillan and Creelman 2005). The ROC is derived from the distributions of trial-sampled magnitudes of the ACC and the estimated background noise. In this analysis, the noise magnitudes were estimated from a response interval that had no preceding rate change. More specifically, noise magnitudes were the difference of the maximum and minimum within the interval 110 to 10 ms prior to a rate change; this pre-change noise was distant in time from responses to the previous rate change while avoiding (within 10 ms) responses to the subsequent change that may have been smeared in time by the non-causal filtering that was used in the analysis. For each stimulus condition, 500 bootstrap samples of ACC and noise magnitudes were generated, with each peak-to-peak sample selected from the mean of 50 waveforms drawn randomly with replacement (Efron and Tibshirani 1991). An empirical ROC curve was formed to derive *d’* index of sensitivity:

$${d}^{^{\prime}}=\sqrt{2}\times z\left(AUC\right)$$

where AUC (area under the curve) gives the proportion of correct discrimination and *z* is the transform to standard deviates. In cases when *AUC* was 0.0 or 1.0 such that *z*(*AUC*) was undefined, the AUC was changed to 1/2* N* or 1–1/2* N*, respectively, where *N* = 500. These adjustments made the possible minimum and maximum *d’* =  ± 4.37. ACC responses having *d’* ≥ 1 (at least one standard deviation from the noise) were considered significant. The advantage of *d’* as a measure of sensitivity of the ACC is that it is dimensionless and can be compared to psychophysical sensitivity.

#### FFR Analysis

The FFR was measured from the same blocks of waveforms as those that yielded the ACC. Waveforms were bandpass filtered between 50 and 3000 Hz using the same filter design as described for the ACC analysis. As with the ACC, the average over the two active electrode channels was computed for analysis. The waveforms were segmented from 0 to 1000 ms with respect to the onset of each base-rate and to each higher-rate pulse train; there were no direct-current corrections of the waveforms. This resulted in an equal number of 150 waveforms for 18 unique pulse rates. Responses to base pulse rates were recorded in both the 36 % and 66 % rate-change conditions. For that reason, only responses to base rates recorded in the 66 % condition were analyzed. The waveforms were then screened for excessive noise as described for the ACC but with an analysis interval of 0-to-1000 ms. In some cases, a small random selection of waveforms (*n* < 7) was then removed to equalize waveform numbers for − and + stimulus polarities. These procedures resulted in an average of 4.8 % rejected waveforms.

The remaining ~ 142 waveforms for each pulse rate were averaged. Hann-windowed ramps with 50 ms onset/offset durations were applied to reduce any residual response to the previous pulse train following a rate change. The fast Fourier transform (FFT) was computed for each waveform to obtain the complex spectra as functions of frequency with 1-Hz resolution. The spectral amplitude representations contained multiple peaks corresponding to the stimulus pulse rate and its higher harmonics, which reflected the fact the FFR waveforms were not sinusoidal. To estimate the overall strength of temporally encoded information distributed across these spectral peaks, the composite FFR was quantified by summing the spectral amplitudes at the pulse-rate frequency and its first four harmonics. The spectral noise floor level was estimated by averaging the amplitudes of twelve spectral bins (six on each side) obtained at each peak and summing the resulting values. Phase values were analyzed only at frequencies corresponding to each pulse rate. FFR latency was estimated by the group delay, which was derived from the linear slope of the phase values unwrapped over frequency (Picton et al. 2003). Selected phase values in three cats (maximum of 2 per cat) were excluded to enable successful unwrapping.

The one-sample Hotelling T^2^ was used to test whether FFR responses in individual cats showed significant synchronized activity compared to the non-synchronized neural background (Hofmann and Wouters 2012; Hotelling 1931; Picton et al. 1987, 2003). For each pulse rate, this multivariate test compared the average real and imaginary components of the complex response against the variation of these components across trials. Significance was assessed at the level of *p* < 0.05. The significance of each composite FFR was also assessed at the group level. Composite FFR amplitudes were first normalized by subtracting the corresponding FFR noise estimates and then compared against a value of zero using a two-tailed one-sample *t*-test. Under the null hypothesis, this noise-subtracted FFR would tend towards zero, given that in the absence of a response, the FFR amplitude at a given frequency would be similar to the noise amplitudes estimated from adjacent spectral bins (Mouraux et al. 2011; Nozaradan et al. 2017).

### Group-Level Statistical Analysis

Group-level analyses of the behavior, ACC, and FFR were performed with repeated measures analysis of variance (RMANOVA, *ranova* function in MATLAB). Main effects and interactions were tested for the factors base rate, rate-change size (66 vs. 36 %), and rate-change direction (decreasing vs. increasing) as appropriate to each experimental design. Significance was assessed at the level of *p* < 0.05 with the Greenhouse–Geisser correction applied for non-sphericity, although the original degrees of freedom are reported. Post hoc comparisons of the psychophysical and ACC results used two-tailed paired-sample *t-*tests to assess whether individual base rates differed significantly by 66 vs. 36 % change size. Whenever appropriate, a false discovery rate (FDR) correction was applied to account for multiple comparisons (Benjamini and Hochberg 1995; Genovese et al. 2002).

## Results

### Psychophysics

**Fig. 3:**
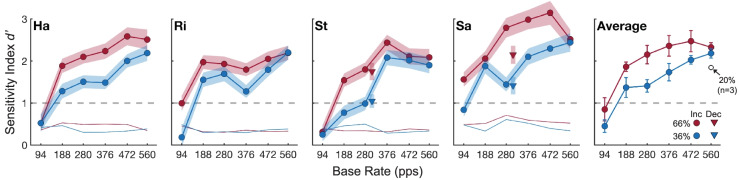
*d* ‘ sensitivity values for individual cats (four left panels) and the group average over cats (right panel). Lines with filled circle symbols show *d* ‘ as a function of the base rate for rate increase of 66 % (red) and 36 % (blue); symbols are labeled “Inc” in the figure legend. Thin colored lines denote chance performance levels for each change size as described in the text. The dashed gray lines denote the threshold criterion, *d* ‘ ≥ 1, for detecting a rate change. Triangle symbols for the cats St and Sa show *d*’ for conditions in which the presentation order of the base and higher rates was reversed to produce a decreasing-rate change; symbols are labeled “Dec” in the figure legend. Shaded regions (four left panels) are the bootstrapped standard errors calculated for each cat and stimulus condition. Error bars (right panel) are the standard error of the mean calculated across the 4 cats for each stimulus condition. The open circle in the right panel shows *d*’ averaged over three cats that completed a supplemental 20 % rate-increase condition. See Tables 1 and 3 for the exact base and higher pulse rate values

The four trained cats performed the hold-release psychophysical task reliably. Figure 3 shows the sensitivity index, *d’*, for individual cats (circle symbols with bootstrapped standard errors, four left panels) and the group average (with standard errors, right panel); thin lines denote chance performance levels, which were derived from the permutation routine described in the Methods. Among cats, performance was above chance level in all but three instances (all at 94-pps base rates), and each cat surpassed the threshold criterion, *d’* ≥ 1 (dashed gray line), for detecting the rate change in all conditions at base rates 188 pps and higher (376 pps and higher for the 36 % change size for cat St). Only one cat, SA, showed *d*’ > 1 for the lowest base rate 94 pps and only for the larger 66 % change size. Cats typically achieved maximum *d’* values around 2 to 2.5, except for cat Sa that showed comparatively greater sensitivity across all conditions, with a maximum *d’* of ~ 3.

Cats showed consistent patterns of performance that were reflected in the group average (Fig. 3, right panel). As expected, *d’* was generally higher for the 66 % compared to the 36 % rate increases, which was reflected by a significant main effect of change size (*F*(1,3) = 45.49, *p* = 0.0067). Among individual base rates, however, *d*’ was significantly greater for the 66 % change than for the 36 % change only at 376 pps (*t*(3) = 5.35, *p* = 0.0128, FDR corrected), and performance for 36 and 66 % changes tended to converge at the highest base rate of 560 pps (described further below). Across base rates, performance consistently was the poorest at the lowest rate of 94 pps, with *d’* values near or below 1, increased to maximum *d’* values among the mid-to-high rates, and plateaued or decreased slightly at the highest rates (e.g., ≥ 472 pps). This relationship was reflected by a significant main effect of base rate (*F*(5,15) = 30.32, *p* = 0.0039, Greenhouse–Geisser corrected). There was no significant interaction between base rate and change size (*p* = 0.22)﻿. The increased sensitivity at higher base rates could help to explain the converging *d’* values for 36 and 66 % change sizes observed in several cats at 472 to 560 pps. That is, regardless of the change size, cats may have approached their “ceiling” sensitivity at those rates given that all cats produced maximum *P*_Hit_ values of 85–96 % for base rates between 472 to 560 pps, whereas *P*_FA_ was constant across change sizes.

Although the present study focused on cats’ sensitivity to increasing pulse rates for reasons of training consistency and experimental time, we also tested in a subset of conditions whether cats could detect complementary changes of decreasing pulse rate. After completing all other psychophysical conditions, two cats, St and Sa, were trained using pulse rates from the 280-pps base-rate conditions (Table 1) but reversed in their order of presentation. That is, pulse trains began at 464 pps and decreased to 280 pps (i.e., the 66 % change) or began at 380 pps and decreased to 280 pps (i.e., the 36 % change); all other aspects of the task were the same. As shown by triangle symbols in Fig. 3, the cats detected these decreasing-rate changes with *d*’ > 1 in all instances; these data are shown adjacent to the 280-pps increasing-rate changes. Moreover, sensitivity was comparable between corresponding decreasing and increasing changes, except for cat Sa that showed reduced *d*’ to the decreasing-rate 66 % condition.

### Psychophysics: Supplemental Conditions at High Pulse Rates

It was somewhat surprising that cats could detect rate changes with high sensitivity up to base rates of 560 pps; for comparison, previous studies in humans have shown that human rate difference limens increase markedly (worse performance) at rates above ~ 500 pps (e.g., Carlyon and Deeks 2002). The rate sensitivity of cats at high base rates might simply mean that cat sensitivity to temporal pitch is optimized at pulse rates higher than predicted based on human performance; indeed, the cat’s audible range is about an octave higher than that of human (Heffner and Heffner 1985). Alternatively, it might be that cats were able to utilize non-temporal (i.e., spectral) cues to aid in rate-change detection. For instance, spectral cues might have contributed to the cats’ performance during rate increases if harmonics became fully, or partially, resolved, especially for narrower auditory filters positioned at the low edge of the stimulus passband (Macherey et al. 2014). We addressed those issues by testing supplemental experimental conditions in three trained cats that had previously completed the standard conditions.

**Fig. 4:**
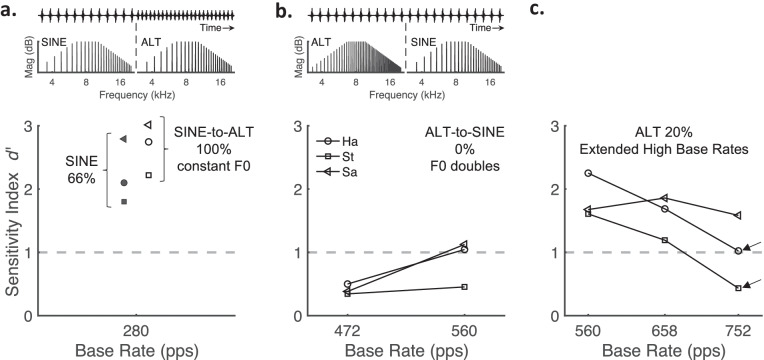
*d* ‘ sensitivity values for supplemental behavioral conditions completed by three cats. Schematics for panels **a** and **b** depict the stimulus designs; the upper parts show a segment of the temporal waveforms (*x*-axes labeled “Time”) with a change in rate and/or phase (vertical dashed lines). The bottom parts show the respective amplitude spectra, before and after the change, for pulse trains that were generated in either SINE or ALT phase (*x*-axes labeled “Frequency (Hz)”). For improved visual representation, the schematics do not depict the actual F0 values used in the experiment and pink noise is not shown but was always present in the stimulus. All panels **a**–**c** show individual cat *d* ‘ values as a function of the base rate (*x*-axes), wherein the threshold criterion (*d* ‘ ≥ 1) is denoted by dashed gray lines. **a** Open symbols show *d* ‘ values for a SINE-to-ALT stimulus that began in SINE phase then changed to ALT phase but always maintained a constant F0 of 280 Hz. This SINE-to-ALT shift doubled the pulse rate (i.e., 100 % rate change), but had a constant amplitude spectrum across the stimulus. For comparison, filled red symbols show *d* ‘ values for the same cats in the standard SINE-phase 66 % rate change condition, as shown in Fig. 3. **b** *d* ‘ values for a ALT-to-SINE stimulus design that began in ALT phase with a given F0 then changed to SINE phase with 2-times the F0 value. This ALT-to-SINE shift had a constant rate (i.e., 0 % rate change) but doubled the harmonic frequencies of the amplitude spectrum. Only base rates of 472 and 560 pps were tested. **c** *d* ‘ values for base rates that extended higher than the standard base-rate conditions (≥ 560 pps) with a 20 % rate increase. Arrows indicate approximate upper limits for detecting a rate change for the cats Ha and St. See Tables 2 and 3 for the exact base- and higher-pulse-rate values used in each supplemental condition

The first supplemental experiment tested whether cats detected temporal stimulus changes in a condition in which spectral cues were certainly absent. In that condition, we generated pulse trains that began in SINE phase with a given F0 then changed to ALT phase with the same F0. This SINE-to-ALT shift produced a 100 % rate change, i.e., doubled the pulse rate, but maintained a constant amplitude spectrum across the stimulus (see Table 2; Schematic Fig. 4a). Figure 4a shows *d’* performance for SINE-to-ALT pulse trains tested at the base rate of 280 pps (open symbols). All cats easily detected the change (*d’* > 2) and in each case, *d’* values were greater than that observed in the standard SINE-to-SINE 66 % condition (filled symbols). This indicates that cats perceived at least as large a pitch change for the 100 % as the 66 % rate increase even though there was no change in amplitude spectrum. On the other hand, if cats had generally relied on resolved spectral cues to perform the task, sensitivity should have declined. This result provides confirmation that, at least up to F0s of 280 Hz, SINE- and ALT-phase stimuli affected the cat’s perception by their temporal rather than spectral properties

**Table 2:** Supplementary psychophysical pulse rate conditions. Exact harmonic complex pulse rates (pps; black text) used for the SINE-to-ALT (left column: 100 % rate increase, constant F0) and ALT-to-SINE (right columns: constant rate, 100 % F0 increase) conditions as described in the text. The fundamental frequency (F0; italicized text) for each harmonic complex is specified below each pulse rate value

Spectral vs. temporal cue test conditions			
	**SINE-to-ALT**	**ALT-to-SINE**	
Base rate (pps) *F0 (Hz)*	280 *280*	472 *236*	560 *280*
+ 100 % pps *+ 0 % F0*	560 *280*		
+ 0 % pps * + 100 % F0*		472 *472*	560 *560*

Secondly, we tested the hypothesis that cats might have utilized resolved spectral cues to perform the task in higher base-rate conditions. In that condition, we generated pulse trains that began in ALT phase then changed to SINE phase with the F0 doubled, hence yielding a 0 % rate increase (see Table 2; Schematic Fig. 4b). This ALT-to-SINE shift would produce no change in pulse rate or, putatively, temporal pitch and could, therefore, only be detected by spectrally resolved harmonics or possibly by timbre differences between ALT- and SINE-phase designs. Figure 4b shows *d’* performance for the ALT-to-SINE pulse trains tested at base rates of 472 and 560 pps. Despite the doubling of F0 and its harmonic frequencies in the stimulus, cats performed well below the *d’* < 1 threshold at the 472-pps base rate. At the 560-pps base rate, two of the three cats performed marginally above threshold (*d’* = 1.05–1.12). Note that, for the 560-pps condition, the SINE-phase harmonics of F0 = 560 Hz would allow for only 1.7 components on average within the estimated cat ERB of 975 Hz centered on 6727.2 Hz (i.e., the lower passband cutoff; see “Methods”), which possibly provided a spectral cue that slightly improved performance. On the other hand, performance was consistently poor for ALT-to-SINE 472 pps, for which at least two SINE-phase harmonics (i.e., spanning 944 Hz) fit within the cat’s 975-Hz ERB, suggesting no spectral or timbre cues were available. By extension, this result argues against a contribution of spectral cues in the standard base-rate conditions, given that the highest F0 presented of 464 Hz (F0 = 280 Hz + 66 %) was lower than those of the present supplemental conditions but produced substantially higher *d*’ values.

Finally, given the cats’ robust performance at the highest standard base rate, we tested whether a limit of temporal pitch sensitivity could be discovered at yet higher base rates (Table 3). Figure 4c shows *d’* measured for base rates of 560, 658, and 752 pps. Rate increases were limited to ~ 20 % to maintain only the 15th and higher harmonics within the stimulus passband. At the 560-pps base pulse rate, *d’* values for the 20 % change were only slightly lower than those for a 36 % increase (also shown in Fig. 3, right panel). At even higher base rates, the cats Ha and St showed declining performance for the 20 % change that dropped near or below *d’* = 1 (1.02, 0.43, respectively) at the highest base rate of 752 pps (see arrows in Fig. 4c). The third cat (Sa) performed above threshold at all base rates for the 20 % change. Notably, this cat was the best performer among the standard rate conditions.

**Table 3:** Supplementary psychophysical pulse rate conditions. Columns specify the exact harmonic complex pulse rates (pps; black text) used for each extended high base-rate condition (top row) and rate increases of 20 % (bottom row). The fundamental frequency (F0; italicized text) for each harmonic complex is specified below each pulse rate value

Extended base-rate conditions			
	**ALT Phase**		
Base rate (pps) *F0 (Hz)*	560 *280*	658 *329*	752 *376*
+ 20 % *F0*	672 *336*	790 *395*	902 *451*

### ACC

**Fig. 5:**
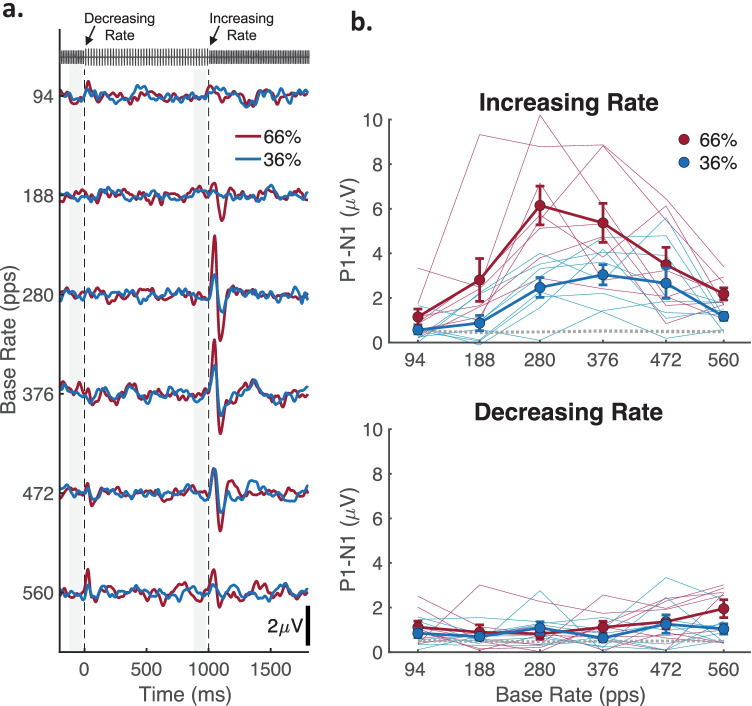
Scalp-recorded ACC waveforms and magnitudes. **a** Grand average ACC waveforms recorded for acoustic pulse trains that continuously alternated between the base and higher rates every 1 s. The *x*-axis indicates the epoch timeline from 200 ms before to 1800 ms after the decreasing-rate change, such that decreasing- and increasing-rate changes are represented at 0 and 1000 ms, respectively (vertical dashed lines). Rate changes are depicted by the example pulse train at the top of the panel; as in psychophysics, higher-rate pulse trains were reduced in level to maintain constant RMS levels with that of the base rate. ACC waveforms for six base-rate conditions are distributed along the *y*-axis for rate changes of 36 % (blue lines) and 66 % (red lines). **b** Filled symbols show the group mean ACC magnitude quantified by the P1, N1 amplitude differences as a function of base rate for rate changes of 36 % (blue) and 66 % (red). Error bars are the standard error of the mean across *N* = 8 cats. The top and bottom panels show ACC magnitudes for the increasing- and decreasing-rate ACC, respectively

Figure 5a shows grand average ACC waveforms across the six base-rate conditions for rate changes of 36 % (blue lines) and 66 % (red lines). The timeline shows − 200 to 1800 ms, with the rate decreases and increases represented at 0 and 1000 ms, respectively. Increasing-rate ACC waveforms were prominent across most base-rate conditions and were characterized by visible P1 and N1 peaks occurring approximately 50 and 100 ms, respectively, after the rate change. These ACC peaks were larger in amplitude for the 66% than the 36% changes and were larger overall for the mid-to-high base rates of 280 to 472 pps, while being reduced or absent at both the highest (560 pps) and, interestingly, the lowest base rates (< 188 pps). Decreasing-rate ACC waveforms were comparatively small or absent across all base-rate conditions but were somewhat visible at the highest base rates (472 and 560 pps).

Figure 5b shows the ACC magnitude given by the P1-N1 amplitude difference for individual cats (thin lines) and the group averaged (thick lines with standard error bars). Increasing-rate ACCs (upper panel) and decreasing-rate ACCs (lower panel) showed marked differences in overall amplitude and response patterns. Increasing-rate ACCs were typically well above the noise floor (dashed lines) but varied widely among individual cats. In contrast, decreasing-rate ACCs were mostly near or below the noise floor (dashed lines), with a possible exception of the highest base rate of 560 pps. An informal test suggested that the variation in increasing-rate ACC was not related to the age or training status of the cats: the two youngest cats (~ 18 months) in the group were trained and ranked 6/8 and 7/8 in largest ACC amplitudes whereas the largest amplitude (8/8) was produced by the oldest cat (~ 60 months); that cat, with the largest amplitude, was untrained, whereas the other two trained cats were intermediate in age (~ 30 months) and ranked low in amplitude (1/8 and 3/8). Therefore, the variation was more likely due to “nuisance” factors such as electrode placement, head and brain geometry, or anesthesia levels.

Despite the variation in ACC magnitude among cats, there were considerable similarities. First, the large overall differences between increasing- and decreasing-rate ACC were confirmed by a highly significant main effect of change direction (*F*(1,7) = 56.94, *p* = 0.00013; 3-way RMANOVA: change direction × change size × base rate). Subsequent analyses were therefore performed separately for each change direction (i.e., 2-way RMANOVA: change size × base rate). For increasing-rate ACCs, the 66 % rate change produced larger responses than the 36 % change in 85.4 % of all cats and rates, and the largest magnitudes for all cats occurred at mid-to-high base rates (66 % base rate: median = 328 pps, range = 188 to 472 pps; 36 % base rate: median = 376 pps, range = 280 to 472 pps). These patterns were reflected by significant main effects of both 66 % vs. 36 % change size (*F*(1,7) = 26.36, *p* = 0.0013) and base rate (*F*(5, 35) = 13.92, *p* = 0.0000090, Greenhouse–Geisser corrected). An interaction of change size and base rate, however, was also significant (*F*(5,35) = 4.47, *p* = 0.01468, Greenhouse–Geisser corrected), which was likely due to varying magnitude differences between 36 and 66 % responses among individual base rates. Specifically, increasing-rate ACCs were significantly greater for the 66 % change only at 280 and 376 pps (36 vs. 66 %: *t*(7) = 3.42–6.30, *p* = 0.0112–0.00040, FDR corrected). For decreasing-rate ACCs, there was no significant main effect of base rate (*p* = 0.0560, Greenhouse–Geisser corrected), but there was a significant main effect of change size (*F*(1,7) = 8.03, *p* = 0.0253), suggesting the decreasing-rate ACC were somewhat sensitive to the larger pitch reductions. This sensitivity, however, was not reflected significantly among individual base rates after correcting for multiple comparisons (36 vs. 66%: *p* = 0.80–0.042, FDR corrected).

**Fig. 6:**
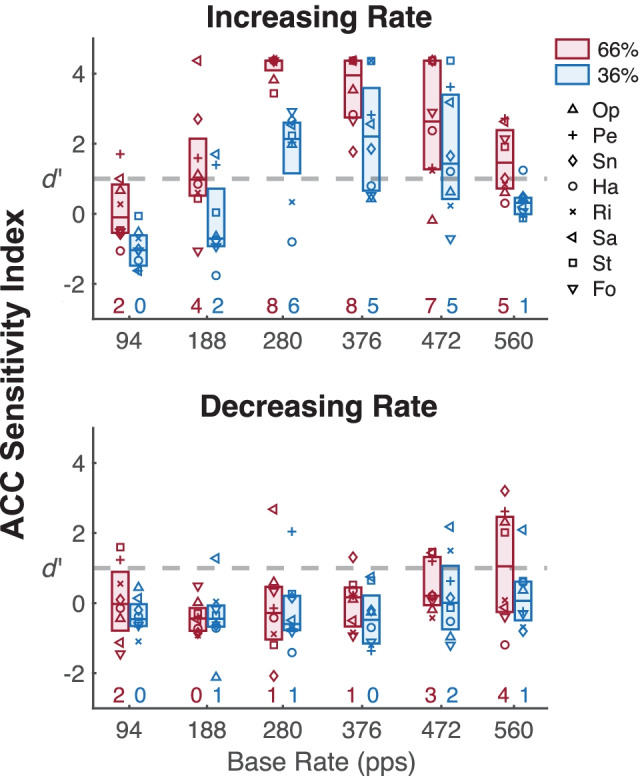
Distributions of ACC *d ‘* values for individual cats. Various symbols show *d ‘* values for each cat as a function of base rate for rate changes of 36 % (blue) and 66 % (red). Boxplots indicate the 25th and 75th quartiles and median *d’* values. Numbers along the abscissa indicate how many of the eight cats produced significant ACCs of *d’* ≥ 1 (horizontal dashed gray line). The top and bottom panels show ACC *d ‘* values for the increasing- and decreasing-rate ACC, respectively

To measure ACC sensitivity on an individual basis, *d’* was computed for each cat from the distributions of bootstrap trial-sampled ACC magnitudes and “pre-change” background noise. Figure 6 shows individual cat *d’* values for increasing- and decreasing-rate ACCs (top and bottom panels, respectively) to the 66 % and 36 % changes (red and blue, respectively). For each condition, boxplots indicate the 25th and 75th quartiles and median *d’* values, while numbers along the abscissa indicate the numbers of cats out of eight that produced significant ACCs of *d’* ≥ 1. For increasing-rate ACCs, *d’* values tended to exhibit a bandpass characteristic such that the number of cats with significant ACCs was the lowest at 94 pps, increased to maxima between 280 to 376 pps, then decreased at 560 pps. For the 66 % rate change, half of the cats produced supra-threshold levels of ACC at 188 pps, which dropped to only 2 cats at 94 pps (0 cats for the 36 % change), suggesting there was lower limit of detection at these rates, below which the ACC was insensitive to rate changes. Although *d’* was also consistently reduced at the highest rate of 560 pps, the ACC was still above threshold in 5 of 8 cats for the 66 % rate change. For decreasing-rate ACCs, fewer than half of the cats showed significant *d*’ values for any condition, with one exception being the 66 % decrease at 560 pps for which half the cats had *d*’ values > 1. This result was consistent with the increasing-rate ACC in suggesting that rate-change detection in some cats may extend to base rates of 560 pps or higher. Note that Cat Sa, which tended to show the best performance in behavioral detection of increasing pulse rates, consistently had among the highest ACC *d* ‘ values for increasing-rate changes.

ACC latencies were assessed for the individual P1 and N1 peaks but failed to show any substantial stimulus-related patterns. Latency values for the increasing-rate changes showed no significant effects of base rate or change size (*p* > 0.1). Decreasing-rate change latencies were deemed unreliable due to the consistent lack of significant amplitude responses.

#### Comparison of ACC with Psychophysics

**Fig. 7:**
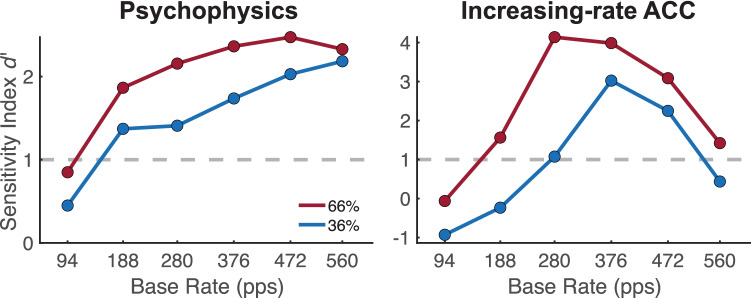
Comparison of psychophysical and ACC sensitivity in the four psychophysically trained cats. **a** Average *d’* values are shown for psychophysical performance (left panel) and the increasing-rate ACC (right panel) as a function of the base rate for rate increases of 36 % (blue) and 66 % (red). To aid comparisons between measures, the vertical axes are scaled so that the maximum *d’* and the threshold values (dashed gray lines, *d’* = 1) had the same position across the panels

Figure 7 shows *d’* for the psychophysical results (left panel) and the increasing-rate ACC (right panel) averaged over the four cats that were evaluated in both procedures. The range of *d’* values varied between measures, which was likely due to differing properties of the underlying response distributions (e.g., *P*_Hit_ vs. N1-P1 magnitudes). For that reason, to facilitate comparisons, the vertical axes were scaled so that the maximum *d’* values and the threshold (*d’* = 1) for each measure had the same position across the panels. Relative to the threshold, the ACC showed similar overall characteristics to the psychophysics. More specifically, *d’* values among the 66 % changes were < 1 at the base rate of 94 pps and were > 1 at all other base rates. The measures differed, however, in their sensitivity at higher base rates. The ACC responses were highly reduced at base rates greater than 376 pps compared to behavioral sensitivity, which remained elevated at those rates. Moreover, the ACC was less sensitive to the 36 % change size, showing responses that were below threshold in three base-rate conditions (94, 188, 560 pps) compared to just 94 pps in the psychophysics.

### ACC: Supplemental Conditions

#### Empirical Evaluation of the Contribution of Loudness Cues to the ACC

The strong preference of the ACC for increases compared to decreases in pulse rate was unexpected, although it resembled the finding by Presacco and Middlebrooks (2018) that ACCs were typically evoked by increases, but not decreases, in pure-tone sound pressure level. Although the RMS level of the higher-rate pulse train in the present study was always matched to that of the base rates, it was in principle possible that ACCs were evoked, in part, by a residual increase in loudness for the higher rates, thereby producing similar asymmetrical response patterns as observed for pure-tone level changes. To test this hypothesis, we performed two supplemental ACC experiments that evaluated the effect of level cues superimposed with the changes in pulse rate. Four cats from the standard ACC experiment were tested. Both experiments utilized only the pulse rates from ALT-phase 376 pps base-rate condition and its 66 % higher rate of 624 pps, as these pulse rates produced a strong increasing-rate ACC and were unlikely to involve resolved harmonics due to their low F0 values of 188 and 312 Hz, respectively. Unless otherwise stated, all procedures were the same as for the standard ACC experiment.

**Fig. 8:**
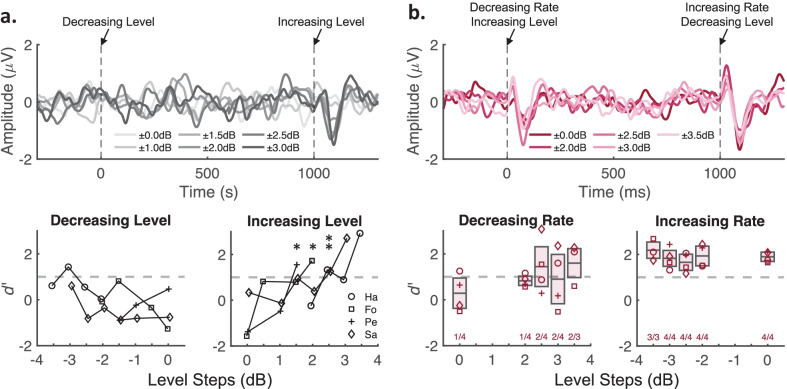
Scalp-recorded waveforms and *d ‘* values for supplemental ACC experiments to evaluate the contribution of loudness cues to the ACC. **a** Experiment that evaluated the minimal sound pressure level difference (i.e., level threshold) that can evoke the ACC for a constant-rate pulse train of 624 pps. Top panel: average ACC waveforms for pulse trains that alternated in level between 65 dB SPL and various increasing-level steps (shades of gray; level steps indicate 65 dB SPL plus 0 to 3.5 dB). Formatting details are the same as Fig. 5a, but with level decreases and increases represented at 0 and 1000 ms, respectively (vertical dashed lines). Bottom panels: ACC *d ‘* values for the series of level steps presented to each cat (various symbols) shown separately for level decreases (bottom-left panel) and increases (bottom-right panel). Asterisks indicate the ACC level threshold determined for each cat. **b** Experiment to evaluate whether increasing loudness cues are concomitant with increasing pulse rates by reducing the higher-rate level relative to that of the base rate. Top panels: average ACC waveforms for pulse trains that alternated between 376 pps and the 66 % higher rate while the higher-rate level was reduced by various level steps (shades of red; level steps indicate 65 dB SPL minus 0 to 3.5 dB). Formatting details are the same as Fig. 5a, but with decreasing-rate/increasing-level changes and increasing-rate/decreasing-level changes represented at 0 and 1000 ms, respectively (vertical dashed lines). Bottom panels: ACC *d ‘* values for the level steps presented to each cat (various symbols) shown separately for rate decreases (bottom-left panel) and increases (bottom-right panel). Boxplots indicate the 25th and 75th quartiles and median *d’* values. Numbers along the abscissa indicate the numbers of cats out of three or four that produced significant ACCs. **a** and **b** The horizontal dashed gray lines (bottom panels) denote the threshold criterion, *d* ‘ ≥ 1, for a significant ACC response

The first experiment estimated the minimal level difference that could evoke the ACC for a pulse train having a constant rate. Level changes were introduced to the 624 pps pulse train (i.e., the higher rate) that was putatively associated with an increasing loudness cue in the standard ACC experiment. Over successive experimental runs, a level step was increased by 0.5 or 1.0 dB until an ACC was clearly present; the minimum step was 0 dB (i.e., no change) and the maximum step tested was 3.5 dB. For each of those conditions, the pulse train began with the original sound pressure level of 65 dB SPL for 1 s, switched to a higher level for 1 s (e.g., 67 dB SPL), then decreased to the original level and so on for each 12 s block, all at a constant pulse rate. The top panel of Fig. 8a shows the averaged ACC waveforms for each level change (shades of gray), wherein the decreasing- and increasing-level changes are represented, respectively, at 0 and 1000 ms; note, average waveforms are only shown for conditions in which 2 or more cats were tested. ACC morphologies were not present for the decreasing levels but were visible for various increasing-level steps. The bottom panels of Fig. 8a show the associated ACC *d*’ values for the series of level steps presented to each cat. The results were comparable to the previous finding for pure-tone level changes (Presacco and Middlebrooks 2018). Decreasing-level steps (Fig. 8a, lower left panel) failed to produce significant ACCs (i.e., *d*’ > 1) for any level reduction, except for cat HA for a − 3 dB change. Increasing-level steps (Fig. 8a, lower right panel), however, tended to produce significant ACCs in all cats for level steps greater than + 1 dB, above which the ACC threshold ranged among cats from + 1.5 to + 2.5 dB with a median of + 2.25 dB (denoted by asterisks).

The second experiment tested whether the putative increasing-loudness cue would be removed by reducing the level of the higher pulse rate relative to that of the base rate. The presence of the loudness cue could, therefore, be inferred only if the increasing-rate ACC was diminished or eliminated by this level reduction procedure. An initial experimental condition replicated the standard ACC experiment by matching the higher-pulse-rate RMS to that of the base rate, i.e., 0 dB RMS difference between pulse trains. Next, based on the observed ACC thresholds for a level-only change (Fig. 8a, i.e., ~ 2.25 dB), the higher rate was reduced by level steps of − 2 to − 3.5 dB in intervals of 0.5 dB assigned randomly across separate experimental runs. In each of those conditions, the pulse train began with the base rate at 65 dB SPL for 1 s, increased in rate and decreased in level (e.g., 63 dB SPL) for 1 s, then returned the base rate and level, and so on for each 12 s block. One cat (Pe) was unable to complete the − 3.5 dB step due to weakened anesthesia levels. The top panel of Fig. 8b shows the grand mean average waveforms across cats. ACC morphologies were visible for both the decreasing-rate (0 ms) and increasing-rate (1000 ms) changes across various level steps (shades of red). When no level adjustment was made (0 dB), ACC *d’* values were comparable to the standard ACC experiment insofar as the decreasing-rate ACC in most cats was not significant (Fig. 8b, lower-left panel, *d*’ > 1 in 1/4 cats), whereas the increasing-rate ACC was significant in all cats (Fig. 8b lower-right panel). Consistent with the level-only change results (Fig. 8a), increasing-level steps for the base pulse rate tended to produce higher *d’* values (non-significant *t*-tests, 0 dB vs. + 2.0, + 2.5, + 3.0, or + 3.5 dB, *p* > 0.10). Importantly, contrary to the hypothesis that an increase in loudness contributed to the increasing-rate ACC, *d’* values were not significantly reduced for decreasing-level steps applied to the higher pulse rate (*t*-test, 0 dB vs. − 2.0, − 2.5, − 3.0, or − 3.5 dB, *p* > 0.40), nor did any increasing-rate ACC fall below *d’* = 1. These results suggest it was unlikely that differences in ACC responses between decreasing- and increasing-rate changes were dependent on loudness cues in the pulse trains.

### FFR

**Fig. 9:**
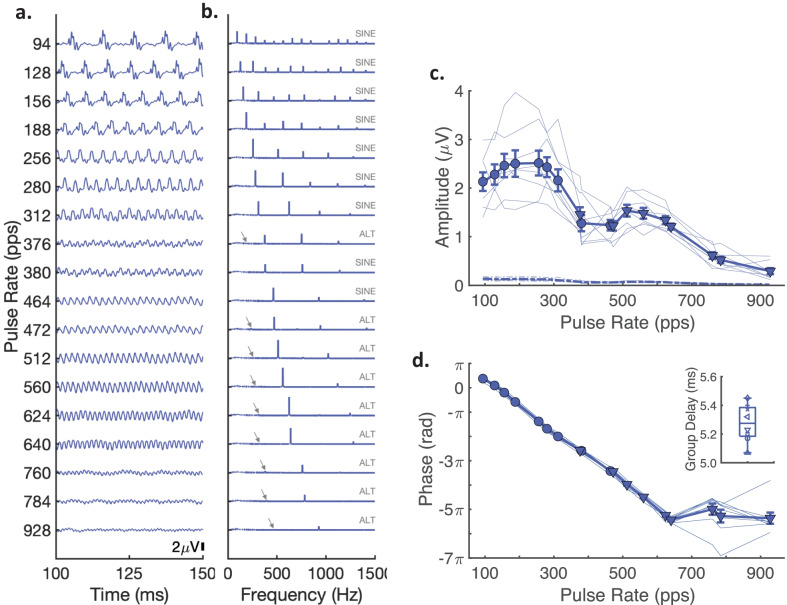
Scalp-recorded FFR temporal waveforms, spectra, composite amplitudes, and phase values. **a** Grand average FFR temporal waveforms and **b** amplitude spectra for each pulse train rate are distributed along the *y*-axes. To aid visual comparison between pulse rates, the temporal waveforms are shown only for a time segment of 100-to-150 ms of the total 1000 ms epoch and amplitude spectra are shown only for frequencies between 0 and 1500 Hz. Gray text above each FFR spectrum indicates whether the pulse train was generated in SINE or ALT phase and gray arrows indicate the F0 frequency value for ALT-phase conditions. **c** The composite FFR amplitudes (i.e., the sum of FFR amplitudes at the pulse rate and its first four harmonics) as a function of pulse rate for individual cats (thin lines) and the group average (thick lines with filled circles). Corresponding dot-dashed lines show the estimated FFR noise floor amplitudes. **d** Unwrapped phase values in radians extracted at each pulse rate frequency for individual cats (thin lines) and the group average (thick line). The inset shows the group delay values computed from the unwrapped phase slope between 94 and 640 pps for each cat (various symbols). The boxplot indicates the minimum and maximum group delay values (whiskers), the 25th and 75th quartiles, and median group delay value

Figure 9a and b show, respectively, the grand-average FFR temporal waveforms and amplitude spectra across 18 pulse-rate conditions. To aid visual comparison between pulse rates, the temporal waveforms are shown only for a time segment of 100-to-150 ms of the total 1000 ms epoch. Temporal responses across all conditions exhibited a visible periodicity corresponding to the respective stimulus pulse rate. Periodic responses at the lower pulse rates, (e.g., < 256 pps) contained multi-peaked structures comparable to auditory brainstem responses (ABR) to single acoustic clicks in cats (Achor and Starr 1980; Jewett 1970). At higher pulse rates, waveforms tended to become more sinusoidal, possibly due the interference patterns of individual responses at shorter inter-pulse intervals (e.g., Wang et al. 2021). The amplitude spectra showed distinct peaks that corresponded to the pulse rate but also contained peaks at several higher harmonics.

Importantly, the responses to the ALT-phase stimuli (≥ 376 pps; see gray labels) showed strong spectral peaks at the pulse rates (2 × F0) and their harmonics, rather than the F0 frequency (indicated by gray arrows). Nevertheless, it was found that small spectral peaks at the F0 of ALT-phase stimuli were significantly present when compared to noise levels at adjacent spectral bins (*t*(7) = 3.40–7.73, *p* = 0.0115–0.00011). These Alt-phase F0 amplitudes, however, were in all cases significantly smaller than the corresponding 2 × F0 pulse rate amplitude (*t*(7) = 3.38–24.92, *p* = 0.0118–0.000000040) by factors ranging from 4.3 to 33.3. It is notable that amplitudes were larger at the higher 2 × F0 frequency even though FFR amplitudes generally decline at higher frequencies. Together, these results validate that the FFR predominantly reflects the pitch-relevant envelope of unresolved harmonic complexes (Krishnan and Plack 2011; Guérit et al. submitted).

Figure 9c shows the amplitude of the composite FFR (i.e., summed amplitudes of the pulse rate and its first four harmonics) for all stimulus pulse rates. The composite FFR for individual cats (thin lines) and the group average (thick line) was generally larger at the lower pulse rates (e.g., < 312 pps) and decreased up to the highest rate, which produced a significant main effect of pulse rate (*F*(17,102) = 32.83, *p* = 0.000031, Greenhouse–Geisser corrected). The relationship, however, was non-monotonic, with local maxima occurring around 156–256 pps and 512 to 560 pps. Similar non-monotonic patterns, or spectral fine structures, have been described in previous published reports in animals and humans (Gardi et al. 1979; Kuwada et al. 2002; Tichko et al. 2017). On the group level, composite FFRs were significant at all tested pulse rates compared to noise levels estimated at adjacent spectral bins (*t*(*x*) = 4.09–18.88, *p* < 0.0024–0.00000020, FDR corrected). FFR responses analyzed in the complex domain for individual cats were also significant at all pulse rates in all cats (Hotelling T^2^ test, *p* < 0.05).

Figure 9d shows the unwrapped phase across all pulse rates for individual cats (thin lines) and the group average (thick line). Phase was successfully unwrapped up to pulse rates of 640 pps, above which rate separations of ≥ 120 pps caused unwrapping to fail due to phase lags that differed by more than 2π. Group delays were therefore computed by the phase slope from 94 to 640 pps. The boxplot inset in Fig. 9d shows that group delay values were highly consistent across cats; values ranged from 5.07 to 5.45 ms with a median of 5.28 ms. This FFR latency is consistent with those previously reported in cat that were identified with neural generators in the rostral auditory brainstem pathways (Gardi et al. 1979; Smith et al. 1975).

## Discussion

The present study demonstrates the feasibility of psychophysical and non-invasive scalp-recorded electrophysiological methods for studying temporal pitch processing in the cat animal model. Stimuli were trains of bandpass acoustic pulses centered in frequency at 8 kHz. In psychophysical measures, cats reliably detected increasing pulse rates in the absence of cochlear place-of-excitation cues. Perceptual sensitivity was the poorest at low base pulse rates (< 100) and increased up to higher base rates (472 to 560 pps), beyond which a tentative upper limit of sensitivity was reached at a base rate of about 700 pps. Scalp recording in sedated cats demonstrated that the cortical ACC can be evoked by the same purely temporal change in pitch, although ACC magnitudes were overall considerably larger for the increasing- than decreasing-rate changes. The dependence of increasing-rate ACC magnitudes on base rate and change size largely resembled the cat’s perceptual sensitivity at low-to-moderate rates, although they differed at the highest pulse rates tested here. Simultaneous recordings of the FFR showed that all the tested stimulus rates were encoded at the brainstem level by robust neural phase-locking. Here, we discuss each of these findings in comparison with previous studies of temporal pitch processing in humans and animals and consider implications for non-invasive studies of temporal pitch processing in the cat animal model of auditory prostheses.

### Perceptual Sensitivity to Temporal Pitch

Few studies have characterized the cat’s perception of the pitch for complex sounds. In early behavioral studies, it was shown that cats could discriminate the missing F0 of harmonic tones, whereby pitch judgments conformed to changes in F0 independently of the constituent harmonic frequencies (Heffner and Whitfield 1976; Whitfield 1980). Although these studies did not explicitly control spectral and temporal pitch cues, cats were less sensitive to higher-frequency, presumably less-resolved, harmonics than they were to lower harmonics (Chung and Colavita 1976; Heffner and Whitfield 1976). This result suggests that, as in humans, pitch percepts in cats are stronger for stimuli that contain resolved harmonics (Houtsma and Smurzynski 1990; Moore et al. 1985; Ritsma 1967; Shackleton and Carlyon 1994), although an effect of absolute frequency, perhaps related to reduced phase locking at higher frequencies, cannot be ruled out (Gockel et al. 2020). Interestingly, that would differ from recent evidence that other non-primate animals rely predominantly on temporal pitch cues (Ferret: Walker et al. 2019; Gerbil: Klinge and Klump 2010, 2009; Chinchilla: Shofner and Chaney 2013).

We observed some differences and similarities between cats and NH humans in studies that used similar band-limited acoustic pulse trains. Cats typically were sensitive to temporal pitch changes at the base rate 188 pps but their performance declined to below threshold at 94 pps. We recently performed a companion study with human NH listeners using stimuli and tasks that closely paralleled those used here (Guérit et al. submitted). Specifically, in each trial, listeners discriminated between a 750-ms bandpass filtered harmonic complex that had a constant pulse rate and one that alternated between a base and a higher (or lower) rate every 250 ms. Although the rate change in that study occurred faster than those in the present study (250 ms vs. 2600–4800 ms), both studies used bandpass filtered complexes that contained no resolved components and required listeners to detect an instantaneous change in the pulse rate of an ongoing stimulus. This latter distinction is important because previous NH human psychophysical studies have shown that the auditory system integrates the pitch of unresolved complex tones over a long temporal window (Plack and Carlyon 1995; Plack and White 2000), which might blur the response to rate changes in ongoing pulse trains, particularly at lower stimulus rates. We found that the human F0 difference limens (F0DLs) were constant for base rates of 94 pps and higher but increased markedly for a base rate of 48 pps. Hence, the results of Guérit et al. (submitted) and of the present study are consistent with both humans and cats having a lower limit of temporal pitch (e.g., Krumbholz et al. 2000; Pressnitzer et al. 2001), but with that limit being higher for cats than for humans. The lower pitch limit in humans has also been shown to increases for complexes filtered into higher-frequency regions, possibly due to less precise encoding of temporal responses originating from the basal cochlea (Cullen and Long 1986; Kaernbach and Bering 2001; Krumbholz et al. 2000; Middlebrooks and Snyder 2010; Ritsma 1962; Stahl et al. 2016). The study of Guérit et al. (submitted), however, compared two frequency bands, 3365–4755 Hz and 7800–10,800 Hz, one of which was lower and one higher than the passband used here, and observed a similar dependence of F0DL on pulse rate in both cases.

Our results also provide new information on the NH cat’s processing of temporal pitch at high pulse rates. Figure 3 shows that cats maintained or improved their sensitivity to rate changes up to 472–560 pps. Supplementary experimental results (Fig. 4b) provided evidence that place-of-excitation or timbre cues were negligible at these higher rates. Also, the auditory filters at the relevant frequencies are somewhat wider in cats than in human (Guérit et al. 2022a), meaning that cues from resolved harmonics would be weaker in cats. It was, therefore, of particular interest to compare across species the upper limit at which temporal acuity degrades. While estimating this upper limit acoustically is constrained by the effects of cochlear filtering at high pulse rates (e.g., resolved harmonics), human listeners have been shown to perceive differences in acoustic pulse rates up to about 700–800 pps (Macherey et al. 2014). When cats were tested at base rates higher than our standard rates but with a smaller-than-standard rate change (see Fig. 4c), overall performance predictably declined and at least two cats showed evidence for an upper temporal limit of about 700 pps. This finding is consistent with an earlier estimate of the upper pitch limit in cats (Chung and Colvatia 1976), and interestingly, is comparable to the estimates in NH humans, which may suggest that temporal pitch breaks down in both species at similar stimulus rates. On the other hand, the incongruent superior performance of a third cat warrants further investigation to substantiate the upper limit in NH cats.

### Electrophysiology in Sedated Cats: ACC and FFR

The present results build on our previous demonstration of the ACC in sedated cats (Presacco and Middlebrooks 2018). The previous study demonstrated ACC sensitivity to purely spectral changes (around pure-tone base frequencies of 2, 4, 8, and 16 kHz), whereas the present study shows ACC sensitivity to non-spectral temporal pitch changes. Notably, the present stimulus design also differs from many previous human EEG and magnetencephalography (MEG) studies of temporal processing that recorded ACCs either to changes in stimulus rates that were too low to elicit pitch (Okamoto et al. 2009, 2012; Undurraga et al. 2021) or to the onset of periodic pitch from an aperiodic stimulus with minimal place-of-excitation cues (Bidelman and Grall 2014; Chait et al. 2006; Gutschalk et al. 2002, 2004; Han and Dimitrijevic 2015, 2020; Hughes et al. 2014; Krishnan et al. 2012; Krumbholz et al. 2003; Seither-Preisler et al. 2006, 2004). Here, robust ACCs were observed for increasing but not decreasing changes in rate. Those increasing-rate ACCs were similar in latency and morphology to ACCs previously recorded in cat to changes in pure tones but tended to have smaller overall magnitudes; maxima of ~ 10 uV in the present study vs. 20–30 uV in Presacco and Middlebrooks (2018). Decreasing-rate ACCs in the present study were near the noise floor. That finding differed from the results of our companion study (Guérit et al. submitted), which found similar-sized ACCs for increasing- and decreasing-rate changes at all base rates tested, which ranged from 94 to 280 pps. That also differs from the observations that neither cats (Presacco and Middlebrooks 2018) nor humans (Martin and Boothroyd 2000) show a consistent preference of the ACC for the direction of changes in pure-tone frequency. Curiously, cats (Presacco and Middlebrooks 2018) and humans (Martin and Boothroyd 2000) show a strong preference of the ACC for increases in pure-tone sound pressure level compared to decreases in level. Nevertheless, we showed that two cats could behaviorally detect rate decreases when trained in a subset of conditions (Fig. 3, rate decreases from 464 or 380 to 280 pps). Thus, the ACC may reflect a difference in the underlying patterns of neural activation (e.g., synchronization) that support perceptual sensitivity to the different rate-change directions.

The dependence of the increasing-rate ACC on base pulse rate and change size was broadly consistent with the cat’s behavioral sensitivity. For example, in the behaviorally trained cats, both ACC and psychophysical *d’* values for the 66 % change condition were consistently below the threshold at 94 pps and remained above the threshold at all higher base rates (Fig. 7). This finding provides validation for the ACC as an objective measure of pitch sensitivity that can act as a surrogate to perceptual pitch tasks in untrained cats. It should be noted, however, that the ACC underestimated the cat’s behavioral sensitivity at higher base rates, such that *d’* values declined markedly after reaching maxima around 280 to 376 pps. The reason for this discrepancy is unclear, but may it reflect an inherent sensitivity limit of the far-field potential ACC such that higher rates may activate distinct populations of pitch-encoding cortical neurons that are not strongly represented at the scalp.

The present FFR recordings in cats replicated previous reports in humans (Gockel et al. 2015; Krishnan and Plack 2011; Guérit et al. submitted) that showed robust neural phase-locking to the periodicity of unresolved harmonic tones, including the doubling of rate for ALT-phase stimuli. The group delay of ~ 5.3 ms (Fig. 9d) agrees roughly with first-peak FFR latencies in previous cat studies and is consistent with dominant neural sources in the rostral brainstem, including the inferior colliculus (Gardi et al. 1979; Smith et al. 1975). It has been shown conclusively, however, that the FFR reflects the composite phase-locked activities of multiple subcortical and even cortical auditory nuclei, which can contribute differentially to the scalp-recorded potential depending on the stimulus and recording design (Chandrasekaran and Kraus 2010; Coffey et al. 2019). Indeed, the non-monotonic or rippled patterns observed in the cats’ spectral FFR amplitudes (Fig. 9b and c) are consistent with frequency-specific phase interference patterns arising from multiple neural sources responding at varying latencies (Gardi et al. 1979; Kuwada 2002; Tichko and Skoe 2017).

The FFR amplitudes were the strongest at low rates and decreased gradually at higher rates. This characteristic is commonly found across species and can be explained by multiple factors, including reduced phase-locking capacity (i.e., upper limits) at higher stages of the auditory pathways, greater susceptibility at higher rates to temporal imprecision among phase-locked neurons, and low-pass filtering properties of the skull (Gardi et al. 1979; Kuwada 2002; Tichko and Skoe 2017). Nevertheless, the FFR amplitude remained significant across all pulse rates that we tested, indicating that synchronized or isomorphic representations of temporal pitch were transmitted through the brainstem pathways. The range of rates above which the FFR declined (~ 300–600 pps) was also substantially higher than observed with analogous stimuli in humans (~ 200 pps) (Guérit et al. submitted). The strong phase-locked brainstem encoding at low rates contrasted markedly with the cats’ poor behavioral and ACC sensitivity to changes at these rates (< 200 pps). This suggest that the cat’s perceptual acuity may depend on non-isomorphic transformations that occur at the thalamic or cortical levels, such as non-synchronized rate or neural place codes specialized for pitch (Wang 2018). Nevertheless, the FFR provides a non-invasive correlate of the requisite temporal processing prior to the cortex that mediates pitch sensitivity.

### Implications for Auditory Prosthesis Research

The responses to band-pass acoustical pulses in this study demonstrated that cats produce robust behavioral and neural responses to a range of rates of pulses delivered to the basal cochlea that are relevant to temporal pitch perception in electric hearing. The typical human CI users can effectively discriminate rates of pulse trains only up to ~ 300 pps, but this upper limit can vary widely across users and electrodes, ranging from 200 to 700 pps (Kong and Carlyon 2010; Macherey et al. 2011; Townshend et al. 1987). Several outstanding questions regarding the neural basis of this upper limit and its variability can be addressed by applying the non-invasive methods developed here in cats chronically implanted with a CI or other auditory prosthesis.

First, the non-invasive measures can link putative neural limitations identified by invasive neurophysiological studies to the awake cat’s perception. For example, recordings in the cat inferior colliculus (IC) suggest that present-day CIs, typically positioned in the cochlear base, activate high-frequency pathways that exhibit relatively low-temporal-acuity transmission of TFS by electric stimulation (Middlebrooks and Snyder 2010). Psychophysical measures in chronically implanted cats can determine whether this neural limitation is reflected in perceptual sensitivity as compared to the present NH baseline measures in cats. Those conclusions could be supplemented by ACC measures, which may provide an objective measure of temporal pitch sensitivity for non-behaving cats (i.e., untrained cats or during experimental surgery). Complementary information can be provided by the electrically elicited FFR, which can serve as an electrophysiological correlate of phase-locked responses in the IC (e.g., Gransier et al. 2021). Furthermore, in conjunction with psychophysics, these objective measures can provide insights as to what levels of the auditory system, brainstem or cortex, the behaviorally relevant neural limitations arise, and how these covary with differences across cats and electrodes. Parallel perceptual and neural measures in NH and CI human listeners can then provide a basis to relate temporal pitch mechanisms found in the cat to human perception.

Second, the non-invasive measures in this study can facilitate longitudinal studies of the cat animal model in which, unlike human studies, the history of deafness and electric stimulation can be well controlled. Neurophysiological studies in the cat IC show that temporal acuity degrades with months of auditory deprivation following hearing loss (Hancock et al. 2012, 2013; Middlebrooks 2018). On the other hand, chronic stimulation in previously deafened cats revealed neuroplastic changes in the brainstem that partially restored temporal acuity (Snyder et al. 1991, 1995; Vollmer et al. 1999, 2005). These findings parallel human CI studies in which longer durations of deafness were associated with poorer performance in temporal processing tasks (Bierer et al. 2015; Cosentino et al. 2016), whereas the upper limits of temporal pitch improved in the months following first activation of an implant (Carlyon et al. 2019). The detailed time-course of those changes and their neural underpinnings can be studied by psychophysics and electrophysiology over naturalistic time periods in which the cat’s deafness and restored hearing are manipulated.

Finally, the non-invasive measures provide useful tools to evaluate novel modes of auditory prosthesis that are not yet feasible in humans. Middlebrook and Snyder (2010) showed that selective stimulation of the cochlear apex activates a low-frequency, high temporal acuity, and brainstem pathways that improved neural phase locking at the level of the IC. Psychophysical and scalp-recorded measures can assess whether these neural benefits, observed in acutely deafened anesthetized cats, improve temporal pitch for cats chronically implanted with a stimulating device that targets the apical auditory nerve fibers, such as a penetrating auditory nerve electrode array, an auxiliary CI electrode placed at the cochlear apex, or by cochlear optogenetic stimulation (Dieter et al. 2019).
